# Cytotoxic immune responses in the lungs correlate to disease severity in patients with hantavirus infection

**DOI:** 10.1007/s10096-016-2592-1

**Published:** 2016-02-12

**Authors:** J. Rasmuson, J. Pourazar, N. Mohamed, K. Lejon, M. Evander, A. Blomberg, C. Ahlm

**Affiliations:** Department of Clinical Microbiology, Infectious Diseases, Umeå University, 90185 Umeå, Sweden; Department of Public Health and Clinical Medicine, Medicine, Umeå University, Umeå, Sweden; Department of Clinical Microbiology, Virology, Umeå University, Umeå, Sweden; Department of Clinical Microbiology, Immunology, Umeå University, Umeå, Sweden

## Abstract

Hantavirus infections may cause severe and sometime life-threatening lung failure. The pathogenesis is not fully known and there is an urgent need for effective treatment. We aimed to investigate the association between pulmonary viral load and immune responses, and their relation to disease severity. Bronchoscopy with sampling of bronchoalveolar lavage (BAL) fluid was performed in 17 patients with acute Puumala hantavirus infection and 16 healthy volunteers acting as controls. Lymphocyte subsets, granzyme concentrations, and viral load were determined by flow cytometry, enzyme-linked immunosorbent assay (ELISA), and quantitative reverse transcription polymerase chain reaction (RT-PCR), respectively. Analyses of BAL fluid revealed significantly higher numbers of activated CD8^+^ T cells and natural killer (NK) cells, as well as higher concentrations of the cytotoxins granzymes A and B in hantavirus-infected patients, compared to controls. In patients, Puumala hantavirus RNA was detected in 88 % of BAL cell samples and correlated inversely to the T cell response. The magnitude of the pulmonary cytotoxic lymphocyte response correlated to the severity of disease and systemic organ dysfunction, in terms of need for supplemental oxygen treatment, hypotension, and laboratory data indicating renal failure, cardiac dysfunction, vascular leakage, and cell damage. Regulatory T cell numbers were significantly lower in patients compared to controls, and may reflect inadequate immune regulation during hantavirus infection. Hantavirus infection elicits a pronounced cytotoxic lymphocyte response in the lungs. The magnitude of the immune response was associated with disease severity. These results give insights into the pathogenesis and possibilities for new treatments.

## Introduction

Hantaviruses are rodent-borne viruses causing disease worldwide. Infections with American hantaviruses (e.g., Andes and Sin Nombre virus) may lead to hantavirus cardiopulmonary syndrome, presenting with severe cardiopulmonary failure and high mortality, while infections with Asian or European hantaviruses are known to cause hemorrhagic fever with renal syndrome, characterized by coagulopathy and acute renal insufficiency [1–4]. The dichotomy of hantavirus syndromes is not clear cut, as patients infected with European Puumala virus (PUUV) commonly present with lung involvement, indicated by lower respiratory tract symptoms and impaired pulmonary gas diffusion capacity [5–7]. There is currently no effective treatment or vaccine.

The pathogenesis is poorly understood, but vascular dysfunction and intense cytotoxic lymphocyte responses are believed to be, at least partly, responsible for the development of disease manifestations [8–11]. Previous studies of hantavirus-infected patients have revealed expansions of cytotoxic T cells (CTLs) and natural killer (NK) cells in the lungs [5, 12, 13]. The presence of virus in the lungs during hantavirus infection has only been evaluated in post-mortem samples from patients infected with American hantaviruses or in small case series of patients with PUUV infection [12–16]. The relationship between viral load and immune response in the lungs has never been established. We aimed to investigate the pulmonary immune response and viral load in acute hantavirus disease. We hypothesized that a cytotoxic lymphocyte response in the lungs, along with detectable viral RNA, would be associated with disease severity.

## Materials and methods

### Patients and bronchoscopy

We included all hospitalized hantavirus-infected patients at the Department of Infectious Diseases (University Hospital, Umeå, Sweden) during the period from January 2008 to March 2011. A total of 47 patients were admitted, but 30 did not participate due to lack of consent (*n* = 19), short hospitalization (<2 days, *n* = 7), or logistical reasons (*n* = 4). Seventeen patients (11 females; median age 54 years, range 31–69) with acute PUUV infection agreed to bronchoscopy. All but one of the included patients were also part of a study evaluating heart and lung manifestations [6]. One patient had allergic asthma, while the rest were previously lung-healthy. Seven patients were smokers, defined as current smoking (*n* = 4) or smoking cessation within the last two years (*n* = 3). Sixteen healthy gender-, age-, and smoking habit-matched volunteers acted as controls. The study was approved by the regional ethical review board at Umeå University (number 07-162 M). Participants were treated according to the declaration of Helsinki and all gave written informed consent.

Bronchoscopy with sampling of bronchoalveolar lavage (BAL) fluid from the right middle lobe was performed in controls and in patients 6–14 days (median 9 days) post symptom onset, as previously described [5]. To avoid bleeding complications, bronchoscopy was undertaken as soon as platelet numbers were improving and deemed sufficient (>100 × 10^9^/L).

### Analyses of BAL fluid

BAL cell differential counts were performed as previously described [17]. Subsets of bronchoalveolar lymphocytes were determined by flow cytometry using the FACSCalibur system (Becton Dickinson, San Jose, CA). Cells were prepared as previously described [5] and stained with fluorochrome-conjugated monoclonal antibodies, as detailed in Table 1. CD25, CD69, HLA-DR, and NKG2D were used as markers for lymphocyte activation. Up to 80,000 total events were collected per sample.

**Table 1:** Antibodies used for flow cytometry determination of bronchoalveolar lymphocyte subsets

Lymphocyte subset	Antibody ligand (fluorochrome)
T cells (CD3^+^)	CD3 (PerCP)
T helper cells (CD3^+^CD4^+^)	CD3 (PerCP), CD4 (FITC)
Cytotoxic T cells (CD3^+^CD8^+^)	CD3 (PerCP), CD8 (PE)
Natural killer cells (CD3^−^CD16^+^CD56^+^)	CD3 (FITC), CD16 (PE), CD56 (PE)
Regulatory T cells (CD3^+^CD4^+^CD25^bright^CD127^low/-^)	CD3 (APC), CD4 (FITC), CD25 (PE-Cy5), CD127 (PE)
Activated T helper cells (CD3^+^CD4^+^CD25^+^)	CD3 (APC), CD4 (FITC), CD25 (PE)
Activated T helper cells (CD3^+^CD4^+^CD69^+^)	CD3 (APC), CD4 (FITC), CD69 (PE)
Activated T helper cells (CD3^+^CD4^+^HLA-DR^+^)	CD3 (APC), CD4 (FITC), HLA-DR (PE)
Activated cytotoxic T cells (CD3^+^CD8^+^CD25^+^)	CD3 (APC), CD8 (PerCP), CD25 (PE)
Activated cytotoxic T cells (CD3^+^CD8^+^CD69^+^)	CD3 (APC), CD8 (PerCP), CD69 (PE)
Activated cytotoxic T cells (CD3^+^CD8^+^HLA-DR^+^)	CD3 (APC), CD8 (PerCP), HLA-DR (PE)
Activated cytotoxic T cells (CD3^+^CD8^+^NKG2D^+^)	CD3 (PerCP), CD8 (FITC), NKG2D (PE)

*APC* allophycocyanin; *FITC* fluorescein isothiocyanate; *PE* phycoerythrin; *PE-Cy5* phycoerythrin-Cy5; *PerCP* peridinin chlorophyll protein

Antibody clones used were: SK7 (anti-CD3); SK3 (anti-CD4); SK1 (anti-CD8); B73 (anti-CD16); MY31 (anti-CD56); 2A3 (anti-CD25 PE); L78 (anti-CD69); L243 (anti-HLA-DR); 1D11 (anti-NKG2D); BC96 (anti-CD25 PE-Cy5); hIL-7R-M21 (anti-CD127). All antibodies were purchased from Becton Dickinson (San Jose, CA), except anti-CD25 PE-Cy5 (BioLegend, San Diego, CA)

Cytotoxic lymphocyte activity and mediators of inflammation were determined in BAL fluid supernatants using commercial enzyme-linked immunosorbent assay (ELISA) kits; for granzyme A (GzmA) (BioVendor, Brno, Czech Republic), granzyme B (GzmB) (Abcam, Cambridge, MA), and tumor necrosis factor-α and interleukin 6 (R&D Systems, Abingdon, UK).

PUUV RNA was analyzed separately in bronchoalveolar cells and supernatant. Viral RNA was extracted as previously described [18], and cDNA was generated by using 5 μL RNA and the GoScript™ Reverse Transcription System (Promega Biotech, CA), according to the manufacturer’s instructions. Before the polymerase chain reaction (PCR) assay, cDNA was pretreated with HK™ UNG (Epicentre Technologies, Madison, WI) to ensure that any contaminating PCR products did not affect subsequent PCR. The quantitative PCR was performed in triplicate, and including negative controls [18].

### Indicators of disease severity

Clinical data indicating severe disease were need for oxygen treatment, lowest recorded systolic blood pressure, and number of days in hospital. Laboratory investigations in serum or plasma included C-reactive protein, N-terminal pro-B-type natriuretic peptide (NT-proBNP), troponin T, albumin, lactate dehydrogenase (LDH), and creatinine, along with leukocyte and platelet counts, analyzed at the hospital’s accredited laboratory. Samples were taken on the day of study inclusion and then every second day throughout hospitalization, including the day of bronchoscopy.

### Statistical analysis

Statistical analyses were performed using IBM SPSS Statistics (version 22) by Mann–Whitney U tests for group comparisons and Spearman’s ranked correlations test for correlation analysis. All tests were two-tailed and a *p*-value < 0.05 was considered statistically significant.

## Results

### Clinical and laboratory findings

The clinical characteristics and laboratory results are summarized in Table 2. Briefly, all patients displayed typical clinical presentation for PUUV infection and almost two-thirds of the patients experienced respiratory symptoms, such as dyspnea or dry cough. One-third required supplemental oxygen treatment, due to low oxygen saturation (≤92 %) and/or significant dyspnea and one-third showed transient hypotension (systolic blood pressure ≤90 mmHg). A majority of the patients displayed thrombocytopenia, acute renal impairment (elevated creatinine), systemic inflammation (elevated C-reactive protein and leukocytosis), and increased LDH indicating cell damage (Table 2). Albumin concentrations were low, suggesting vascular leakage (Table 2), and were correlated with patients’ lowest recorded systolic blood pressure (*r* = 0.61, *p* = 0.009). Patients requiring oxygen treatment had significantly lower albumin and trends towards higher creatinine and leukocyte counts (Table 3) compared to those not needing oxygen. No patient required dialysis and all survived.

**Table 2:** Clinical characteristics and laboratory results in patients with Puumala hantavirus infection

Days of hospitalization	5 (2–9)
Clinical findings	
Hypotension (≤90 mmHg)	6 (35 %)
Respiratory symptoms	10 (59 %)
Dyspnea	8 (47 %)
Dry cough	5 (29 %)
Oxygen treated	5 (29 %)
Laboratory results	
Leukocyte count (3.5–8.8 × 10^9^/L), max	9.0 (5.3–27.0)
C-reactive protein (<3 mg/L), max	78 (35–249)
Platelet count (145–387 × 10^9^/L), min	63 (18–305)
Creatinine (<105 μmol/L), max	173 (59–1072)
Lactate dehydrogenase (<3.4 μkat/L), max	4.8 (3.8–12.3)
Albumin (36–45 g/L), min	28 (14–34)
NT-proBNP (<150 ng/L), max	1768 (121–8878)
Troponin T (<15 ng/L), max	8 (0–22)

*NT-proBNP* N-terminal pro-B-type natriuretic peptide

Clinical findings are presented as number of patients (%) with the respective finding, while blood laboratory results (reference values) and numbers of days of hospitalization are expressed as median (range)

**Table 3:** Differences in parameters comparing patients with or without need for supplemental oxygen treatment

	Oxygen treatment (*n* = 5)	No oxygen treatment (*n* = 12)	*p*-Value
Laboratory results			
Leukocyte count (10^9^/L), max	15.1 (9.1–15.8)	8.6 (7.0–9.5)	0.058
C-reactive protein (mg/L), max	187 (65–203)	73 (37–128)	0.11
Platelet count (10^9^/L), min	63 (42–85)	63 (42–90)	1.00
Creatinine (μmol/L), max	276 (186–327)	130 (83–219)	0.058
Lactate dehydrogenase (μkat/L), max	4.9 (4.6–6.2)	4.5 (4.2–5.1)	0.21
Albumin (g/L), min	22 (17–27)	29 (24–31)	0.034
NT-proBNP (ng/L), max	2342 (1419–6862)	728 (523–4393)	0.17
Troponin T (ng/L), max	10 (7–19)	8 (0–11)	0.22
Differential cell counts			
Macrophages	22.0 (17.1–44.3)	15.0 (11.4–30.2)	0.21
Eosinophils	0.1 (0–0.2)	0 (0–0.8)	0.21
Neutrophils	0.6 (0.5–0.7)	0.4 (0.2–0.6)	0.14
Lymphocytes	9.9 (6.2–15.7)	3.2 (2.0–7.7)	0.015
Flow cytometry			
T cells	9.5 (5.6–14.9)	2.6 (1.5–7.5)	0.020
T helper cells	1.9 (1.7–5.2)	0.6 (0.3–2.7)	0.079
Cytotoxic T cells	7.3 (3.5–9.3)	1.4 (1.1–4.8)	0.036
Natural killer cells	0.5 (0.4–0.6)	0.2 (0.1–0.5)	0.10
Regulatory T cells	0.6 (0.4–1.5)	0.4 (0.2–0.7)	0.19
T helper cells, CD25^+^	0.1 (0.1–0.2)	0 (0–0.1)	0.047
T helper cells, CD69^+^	0 (0–0)	0 (0–0.3)	0.82
T helper cells, HLA-DR^+^	0.5 (0.3–1.3)	0.1 (0.1–0.4)	0.066
Cytotoxic T cells, CD25^+^	0.1 (0–0.5)	0.1 (0–0.1)	0.40
Cytotoxic T cells, CD69^+^	0 (0–0.2)	0.1 (0–0.2)	0.96
Cytotoxic T cells, HLA-DR^+^	3.6 (1.5–4.1)	0.9 (0.4–2.9)	0.11
Cytotoxic T cells, NKG2D^+^	2.6 (1.0–4.3)	0.6 (0–1.0)	0.020
Granzymes and cytokines			
Granzyme A	326 (104–643)	255 (116–509)	0.83
Granzyme B	33 (6–84)	5 (0–23)	0.070
Interleukin 6	0.8 (0.7–1.8)	0.9 (0.6–1.5)	0.83
Tumor necrosis factor-α	0 (0–0)	0 (0–0.6)	0.24
Viral load	1.4 × 10^1^ (0.2 × 10^1^ to 6.2 × 10^5^)	3.0 × 10^3^ (1.2 × 10^1^ to 1.5 × 10^4^)	0.40

Results are expressed as median (25th–75th percentiles). Laboratory results were obtained by analyses on blood, serum, or plasma. Bronchoalveolar lavage fluid results represent: cells per mL ×10^4^ (×10^2^ for regulatory T cells), pg of granzymes and cytokines per mL, and copy numbers of Puumala virus RNA per 10^4^ bronchoalveolar cells. *p*-Values were determined by the Mann–Whitney U test

### Cytotoxic responses in the lungs

**Fig. 1:**
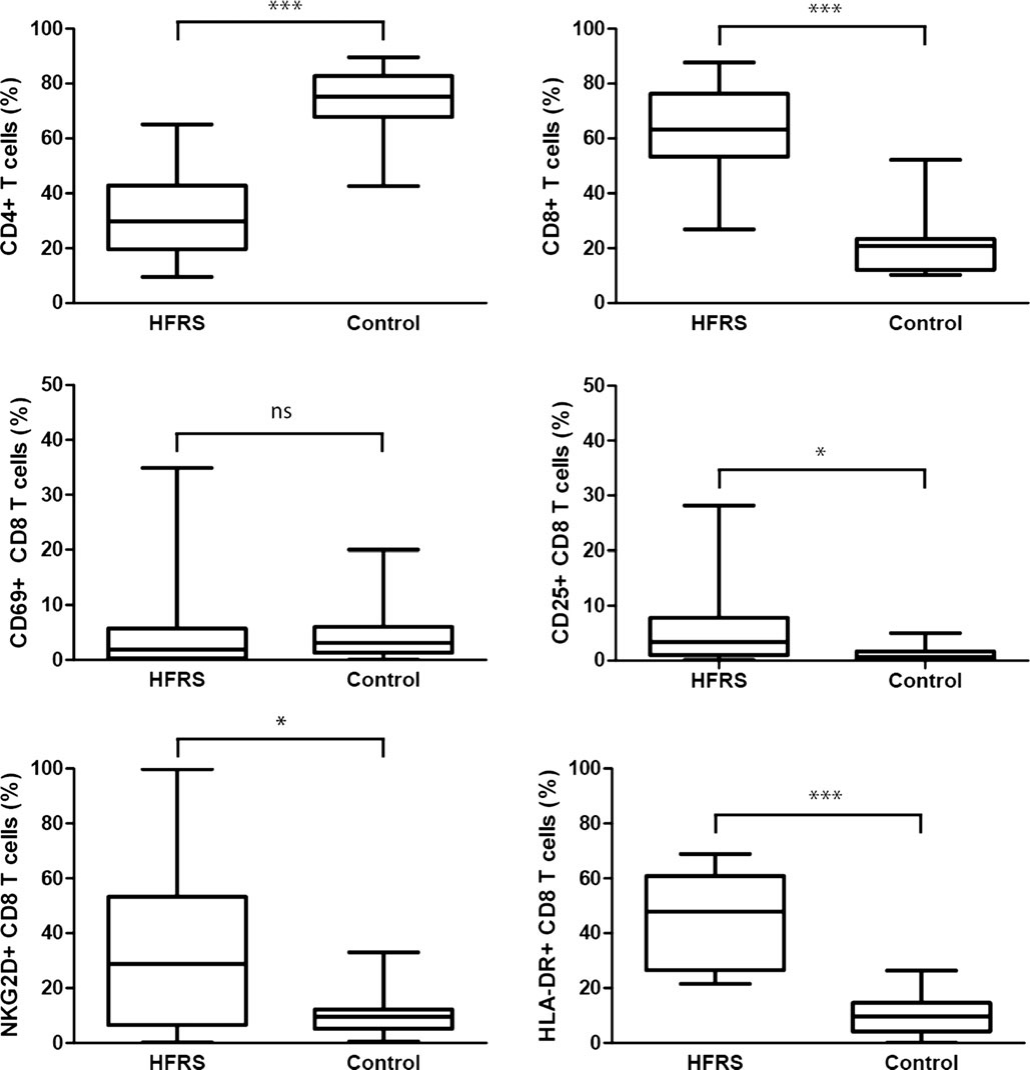
Proportions of bronchoalveolar T cell subsets in patients with acute Puumala hantavirus infection compared to the uninfected healthy controls. Patients displayed an inverse CD4/CD8 T cell ratio due to expansion of the CD8^+^ T cell population. Large proportions of CD8^+^ T cells showed evidence of an active state in hantavirus infection, indicated by significantly higher expression of activation markers CD69, HLA-DR, and NKG2D on CD8^+^ T cells in patients compared to the healthy controls. *p < 0.05, ***p < 0.001 for comparisons by the Mann–Whitney U test, ns = not significant

Higher lymphocyte numbers were found in patients compared to controls (Table 4). The flow cytometry results revealed an inversed bronchoalveolar CD4/CD8 T cell ratio in patients, due to expansions of the CD8^+^ cytotoxic subset, also showing strong activation mainly in terms of frequent HLA-DR and NKG2D expression (Fig. 1). The lymphocyte expansion was, considering absolute numbers, further explained by significantly higher numbers of CTLs and NK cells in patients compared to controls, while the numbers of CD4^+^ T helper (Th) cells were similar (Table 4). The numbers of CTLs expressing activation markers CD25, CD69, HLA-DR, or NKG2D were significantly higher in patients, while no such activation was seen in the Th subset in either relative or absolute numbers (Table 4 and data not shown). Regulatory T (Treg) cells were significantly fewer in patients (Table 4). Although Tregs were few, their number in patients (but not in controls) were significantly correlated to the number of T cells, Th cells, CTLs, and activated CTLs (data not shown), suggesting that stronger T cell responses proportionally also included Tregs. Serum LDH concentration on the day of bronchoscopy correlated to the magnitude of the immune response detected in BAL fluid, expressed as numbers of lymphocytes (*r* = 0.66, *p* = 0.004), T cells (*r* = 0.73, *p* = 0.001), CTLs (*r* = 0.82, *p* < 0.001), and HLA-DR^+^ CTLs (*r* = 0.67, *p* = 0.007).

**Table 4:** Bronchoalveolar immune responses in patients versus healthy controls

	Patients (*n* = 17)	Controls (*n* = 16)	*p*-Value
Return volume	95.0 (81.0–119.0)	108.0 (67.5–123.0)	0.86
Differential cell counts			
Macrophages	16.8 (11.7–33.5)	20.5 (13.4–32.1)	0.67
Neutrophils	0.5 (0.2–0.6)	0.2 (0.0–0.4)	0.088
Eosinophils	0.00 (0.00–0.11)	0.05 (0.00–0.28)	0.40
Lymphocytes	4.2 (2.1–9.3)	1.8 (1.2–2.5)	0.001
Flow cytometry			
T cells	4.3 (1.9–9.1)	1.5 (1.1–1.8)	0.002
T helper cells	1.3 (0.4–2.5)	1.0 (0.6–1.5)	0.71
Cytotoxic T cells	2.6 (1.1–6.4)	0.3 (0.1–0.6)	<0.001
Natural killer cells	0.08 (0.05–0.11)	0.04 (0.02–0.07)	0.034
Regulatory T cells	0.42 (0.22–0.80)	1.38 (0.77–3.14)	0.004
T helper cells, CD69^+^	0.02 (0.00–0.04)	0.01 (0.00–0.07)	0.62
T helper cells, HLA-DR^+^	0.23 (0.06–0.59)	0.20 (0.12–0.32)	0.46
T helper cells, CD25^+^	0.06 (0.02–0.14)	0.06 (0.02–0.14)	0.80
Cytotoxic T cells, CD25^+^	0.06 (0.02–0.19)	0.00 (0.00–0.01)	<0.001
Cytotoxic T cells, CD69^+^	0.05 (0.01–0.16)	0.01 (0.00–0.03)	0.046
Cytotoxic T cells, HLA-DR^+^	1.14 (0.46–3.12)	0.03 (0.01–0.06)	<0.001
Cytotoxic T cells, NKG2D^+^	0.80 (0.06–2.11)	0.04 (0.01–0.05)	0.001
Granzymes and cytokines^a^			
Granzyme A	287.5 (126.3–484.5)	19.7 (13.7–59.4)	<0.001
Granzyme B	6.3 (0–30.4)	0 (0–0)	<0.001
Interleukin 6	0.8 (0.7–1.5)	1.6 (0.9–2.1)	0.08
Tumor necrosis factor-α	0 (0–0)	0 (0–0)	0.61

Results are presented as median (25th–75th percentiles) and represent the number of cells per mL ×10^4^ (×10^2^ for regulatory T cells) or pg of granzymes and cytokines per mL of bronchoalveolar lavage fluid. *p*-Values were determined by the Mann–Whitney U test

^a^Number of subjects with detectable levels; granzyme A (all subjects), granzyme B (12 patients and one control), interleukin 6 (all subjects), tumor necrosis factor-α (three patients and two controls)

Compared to controls, hantavirus-infected patients had significantly higher BAL fluid concentrations of GzmA and GzmB (Table 4), and the two cytotoxins’ levels were correlated (*r* = 0.49, *p* = 0.044). The GzmA and GzmB concentrations in BAL fluid from patients correlated to the numbers of bronchoalveolar lymphocytes (*r* = 0.49, *p* = 0.048 and *r* = 0.57, *p* = 0.016, respectively), T cells (*r* = 0.53, *p* = 0.035 and *r* = 0.62, *p* = 0.011, respectively), CTLs (*r* = 0.58, *p* = 0.018 and *r* = 0.52, *p* = 0.041, respectively), and HLA-DR^+^ CTLs (*r* = 0.62, *p* = 0.013 and *r* = 0.61, *p* = 0.016, respectively). Additionally, GzmB concentrations correlated to the numbers of NKG2D^+^ CTLs (*r* = 0.68, *p* = 0.004). The GzmA concentration in BAL fluid correlated to bronchoscopy-day serum LDH (*r* = 0.54, *p* = 0.025). Interleukin 6 and tumor necrosis factor-α levels in BAL fluid did not differ between patients and controls (Table 4).

### Pulmonary viral load

PUUV RNA could be demonstrated in bronchoalveolar cells in 15 out of 17 (88 %) patients. Four patients also had detectable PUUV RNA in BAL supernatant. When considering positive samples, the median (25th–75th percentiles) viral load was 3.9 × 10^2^ (1.4 × 10^1^ to 1.8 × 10^4^) PUUV RNA copies per 10^4^ bronchoalveolar cells and 4.4 × 10^3^ (1.8 × 10^3^ to 2.6 × 10^5^) copies per mL of supernatant. The viral load in bronchoalveolar cells was inversely correlated to the magnitude of the local immune response, in terms of the numbers of total lymphocytes (*r* = −0.68, *p* = 0.003), T cells (*r* = −0.70, *p* = 0.003), Th cells (*r* = −0.71, *p* = 0.002), CTLs (*r* = −0.71, *p* = 0.002), and HLA-DR expressing CTLs (*r* = −0.58, *p* = 0.023). In addition, the levels of granzymes correlated inversely to viral load, but reaching significance only for GzmA (*r* = −0.69, *p* = 0.002).

### Correlations to disease severity

Pronounced lymphocyte responses in the lungs were found to correlate to several indicators of more severe disease, suggestively reflecting the systemic nature of hantavirus infection. Firstly, significantly higher numbers of bronchoalveolar lymphocytes, T cells, CTLs, and NKG2D^+^ CTLs were found in patients requiring oxygen treatment compared to those without a need for supplemental oxygen (Table 3). Secondly, low systolic blood pressure was associated with an intense bronchoalveolar immune response, in terms of total lymphocytes (*r* = −0.61, *p* = 0.009), T cells (*r* = −0.64, *p* = 0.008), Th cells (*r* = −0.50, *p* = 0.047), CTLs (*r* = −0.57, *p* = 0.022), and NK cells (*r* = −0.58, *p* = 0.019). Thirdly, high numbers of bronchoalveolar CTLs were correlated to laboratory surrogate markers of impaired renal function (maximum creatinine, *r* = 0.54, *p* = 0.030), cardiac dysfunction (maximum NT-proBNP, *r* = 0.67, *p* = 0.004 and maximum troponin T, *r* = 0.41, *p* = 0.023), cell damage (maximum LDH, *r* = 0.56, *p* = 0.025), and vascular leakage (minimum albumin, *r* = −0.50, *p* = 0.049). There were no significant correlations between any indicator of more severe disease and viral load, concentrations of granzymes, Treg cell numbers, or proportions of lymphocyte subsets in BAL fluid (Table 3 and data not shown).

## Discussion

We have demonstrated that hantavirus infection induces an activated cytotoxic effector immune response in the lungs. The magnitude of the immune response was associated with need for oxygen treatment, indicating poor gas exchange, as well as several systemic markers for disease severity.

Hantaviruses cause infections with varying severity of pulmonary involvement, characterized by reduced gas exchange due to interstitial or alveolar edema [3, 6]. The immune response, including cytotoxic lymphocytes (CTLs and NK cells), has been considered to be at least partly responsible for the development of hantavirus disease manifestations [8–11]. Here, expansions of CTLs and NK cells were detected in the lungs of hantavirus-infected patients, as previously reported [5, 12, 13, 19]. The high expression of activation markers on CTLs in hantavirus infection corroborates with previous studies [5, 10] and indicates an activated effector state in these cells [20]. Interestingly, CTLs were not required to cause severe disease in a hamster model for lethal Andes hantavirus infection [21], which could question the role of CTLs in human hantavirus pathogenesis. However, in a recently described macaque model for Sin Nombre hantavirus disease that may better mimic hantavirus infection in humans, expansion of activated CTLs in blood was reported, with the highest CTL proportions occurring during the most severe disease stage [22]. In contrast to previous studies comparing proportions of CTLs in BAL [5] or in blood [23] to disease severity, we report here high absolute CTL numbers in the lungs being associated with several indicators of more severe disease, in accordance with a previous report [9]. Moreover, our finding of higher levels of cardiac dysfunction markers in patients with intense lung immune responses may represent a further indication of secondary heart manifestations in hantavirus disease [6, 24].

Granzymes are effector molecules released from cytotoxic lymphocytes and may be used to measure cytotoxic activity [25]. GzmA and GzmB both induce apoptosis but by different mechanisms [25]. To the best of our knowledge, soluble granzymes have not been evaluated in BAL fluid in respiratory viral infections. Intracellular GzmB has been shown to be expressed in activated CTLs or NK cells in lungs in severe infant respiratory syncytial virus infection and lungs of fatal PUUV cases [12, 26], as well as in blood in human hantavirus infection [10, 27] and in a macaque model [22]. Furthermore, serum LDH has been shown to correlate to markers for apoptosis during hantavirus disease [28]. The high concentrations of BAL fluid GzmA and GzmB found in patients in the current study indicate cytotoxic lymphocyte degranulation within the airways, likely reflecting the killing of hantavirus-infected cells within the airways (immune cells or bronchial epithelial cells). In support of this scenario, we report that bronchoalveolar CTLs, granzymes, viral load, and serum LDH were all significantly correlated. However, a demonstrated hantavirus-conferred resistance to apoptosis of infected cells, with an inability at least for NK cells to clear virus-infected cells, could lead to uninfected bystander cell death, protracted cytotoxic immune responses, and excessive immunopathology [27, 29].

Regulatory T cells are important regulators of the immune system and function to achieve balanced effector responses that clear pathogens without causing excessive immunopathology [30]. High numbers of Treg cells have been shown to maintain persistence and avoid immunopathology in various chronic viral infections, including hantavirus infection in the natural rodent host [31], but little is yet known when it comes to Treg responses in acute human viral infections [30]. Low Treg levels have been proposed to be responsible for the development of West Nile fever [32], severe dengue [33, 34], as well as hantavirus disease in humans [35]. Accordingly, we report bronchoalveolar Tregs being significantly fewer in hantavirus-infected patients compared to controls, suggesting an inadequate Treg cell response, as previously shown in blood in human hantavirus infection [10, 36], as well as in the lungs in the lethal hamster model [37]. A recent study of human hantavirus infection showed that high blood leukocyte count, as well as the level of Treg cell activity, was associated with some markers for disease severity [23]. Similarly, we show that the Treg cells response was proportional to the effector T cell response, even if the Tregs numbers were low and possibly inadequate to maintain balance, leading to overly strong effector T cell responses [35]. Taken together, the importance and function of Treg cells in human hantavirus infections are still poorly understood and warrant further studies.

Puumala hantavirus RNA was detected in bronchoalveolar cells in almost all patients, implying that infection of airway lumen cells is highly frequent. As expected, viral RNA was predominately found within the BAL cells. We did not determine which bronchoalveolar cells contained viral RNA. However, previous studies have shown macrophages and lymphocytes (the two major airway cell populations) to be infected by hantaviruses [12, 13, 15]. The results from the current study showed an inverse relationship between the numbers of CTLs and Th cells and viral load, suggesting a beneficial role for T cells in hantavirus clearance. Based on our data, we could not show any relation between pulmonary viral load and disease severity. Nevertheless, such a relation cannot be excluded and an earlier BAL sampling could, speculatively, have revealed different results.

The study design with bronchoscopy at one single time point is a limitation of the current study. Serial, and earlier, BAL sampling would have given additional information about the time kinetics of the relation between the viral and immunological responses in the lungs. However, this was not feasible, as earlier bronchoscopy was deemed an unacceptable risk due to thrombocytopenia and risk for pulmonary bleeding.

The major strength of the current study is that lymphocyte subsets, viral load, and markers for cytotoxic activity have been investigated simultaneously in the lungs of patients with acute hantavirus infection, resulting in the unique possibility to evaluate the associations between these aspects.

In conclusion, the magnitude of the cytotoxic effector response may determine disease severity in patients with hantavirus infection. Whilst immunomodulatory treatment has not yet been proven to be successful [38], the present results may give valuable insights into the pathogenesis of hantavirus infections and, thereby, open up possibilities to develop new treatments.
